# Limitations in activities of daily living in old age in Germany and the EU – Results from the European Health Interview Survey (EHIS) 2

**DOI:** 10.25646/6226.2

**Published:** 2019-12-11

**Authors:** Beate Gaertner, Markus A. Busch, Christa Scheidt-Nave, Judith Fuchs

**Affiliations:** Robert Koch Institute, Berlin Department of Epidemiology and Health Monitoring

**Keywords:** ACTIVITIES OF DAILY LIVING, GENERAL POPULATION, AGE, EUROPEAN COMPARISON

## Abstract

The health status of older people in Germany can be compared with the health of older people in other European Union (EU) Member States using data on the distribution of limitations in activities of daily living. This concept covers basic limitations in activities of daily living (ADL) such as eating, as well as limitations in instrumental activities of daily living (iADL) such as shopping and managing finances. The second wave of the European Health Interview Survey (EHIS 2) collected data on five ADLs and seven iADLs for people aged 65 or above. An ADL or iADL limitation was defined if a participant reported at least a lot of difficulty in at least one ADL or iADL, respectively. On average, 8.4% of the EU population reported an ADL limitation, with 25.2% reporting an iADL limitation. However, prevalences vary widely between EU Member States and are lower in Germany than the EU average (ADL limitation 6.3%, iADL limitation 14.0%). In general, women, people aged 75 or above, and lower education groups have a higher prevalence of ADL and iADL limitations.

## Introduction

The health status of older people can be described using data on limitations in activities of daily living. These limitations mainly caused by health problems make it difficult or impossible for people to live an independent life. The literature distinguishes between basic activities of daily living (ADL) [1], which includes limitations in eating, mobility – in the sense of being able to get in or out of a bed or chair – and personal care; and instrumental activities of daily living (iADL) [2], such as housework, shopping and managing finances (Info box 1). In accordance with the International Classification of Functioning, Disability and Health (ICF) [3, 4], activities of daily living constitute an important aspect of a person’s functional status.

Limitations in activities of daily living illustrate older people’s care and support needs. Limitations in ADL or iADL are associated with a lower quality of life [6, 7], poorer health [8] and increased mortality [9]. Data on limitations in ADL and iADL, therefore, can be used to demonstrate which population subgroups are particularly affected, and to design prevention and rehabilitation programmes that enable older people to remain independent as long as possible. This is particularly important in the context of demographic change.

Until now, previous European studies have focused on single or selected European Union (EU) Member States, and they used different instruments or different definitions of limitations [10-13]. As such, no data has been available to conduct European-wide comparisons of limitations in activities of daily living for people aged 65 or above. The second wave of the European Health Interview Survey (EHIS 2), therefore, is the first to provide harmonised data from all EU members. Data are primarily used for European standard analyses [14] and can be used for further statistical comparisons, as in this article.

**GEDA 2014/2015-EHIS (for international comparisons)**

**Data holder:** Robert Koch Institute

**Aims:** To provide reliable information about the population’s health status, health behaviour and health care in Germany, with the possibility of a European comparison

**Method:** Questionnaires completed on paper or online

**Population:** People aged 15 years and above with permanent residency in Germany

**Sampling:** Registry office sample; randomly selected individuals from 301 communities in Germany were invited to participate

**Participants:** 24,824 people (13,568 women, 11,256 men)

**Response rate:** 27.6%

**Study period:** November 2014 - July 2015

More information in German is available at www.geda-studie.de and Lange et al. 2017 [17]

## Indicator

As part of the European Health Interview Survey (EHIS) framework, all EU Member States collect data on their population’s health status, health care provision, health determinants and socioeconomic situation (Info box 2).

The EHIS survey focuses on people aged 15 or over living in private households, irrespective of their state of health. In order to achieve a high degree of harmonisation of measurement between the Member States, guidelines on survey methodology and implementation were provided in form of a manual, which also included a sample questionnaire [15]. Data collection for EHIS 2 took place between 2013 and 2015 in all 28 EU Member States. In Germany, EHIS is part of the health monitoring conducted at the Robert Koch Institute, and EHIS 2 has been integrated into the German Health Update (GEDA 2014/2015-EHIS) [16, 17]. A detailed description of the methodology applied in GEDA 2014/2015-EHIS can be found in Lange et al. [17].

Data collection was planned to last for at least three months and include a minimum of one autumn month (September to November). The average length of data collection across all EU Member States was eight months. At the time when EHIS 2 was undertaken, the EU consisted of 28 members. A more detailed description of the methodology applied in EHIS 2 is available in the EHIS quality report [18] and in Hintzpeter et al. [19], which is published in this issue of the Journal of Health Monitoring.

Participants were asked whether they normally faced difficulties when undertaking certain tasks without help. The study focused on five ADLs (eating and drinking, getting in or out of a bed or chair, dressing and undressing, using the toilet, and bathing or showering) and seven iADLs (preparing meals, using the telephone, doing the shopping, managing medication, undertaking light housework, undertaking occasional heavy housework, and organising financial/everyday administrative matters) [20]. The questions were based on Katz et al. [1] and Lawton et al. [2]. Ad Hoc data quality assurance measures not included in standard Eurostat analyses [14] were used. The response categories provided for ADL and iADL were ‘No difficulty’, ‘Some difficulty’, ‘A lot of difficulty’ and ‘Cannot do at all/Unable to do’. For iADL, an additional response category ‘Not applicable (never tried it or do not need to do it)’ was provided, which was recorded as ‘no iADL limitation’ [21]. Moreover, valid data on at least three ADLs or iADLs were required for the identification of an ADL or iADL limitation, respectively. An ADL or iADL limitation was defined as a response indicating that a person faced at least a lot of difficulty conducting at least one ADL or iADL, respectively.

Sociodemographic data on sex, age (age group 65 to 74 and over 75) and education (low, medium and high education group) were collected in accordance with the International Standard Classification of Education (ISCED) 2011 [22].

The analyses are based on data from a total of 79,822 participants (45,657 women, 34,165 men) aged 65 or over from EU Member States. Valid responses were available for 79,014 people on ADL limitations and for 79,054 people on iADL limitations.

**Info box 1:**

**Basic and instrumental activities of daily living (ADL/iADL)**

The International Classification of Functioning, Disability and Health (ICF) defines limitations in activities of daily living as cases where people find it difficult or impossible to perform specific tasks.

To assess limitations in activities of daily living in research and practice, two questionnaires are used that measure limitations in basic activities of daily living (ADL) and limitations in instrumental activities of daily living (iADL).

ADLs comprise the fundamental activities that people have to undertake to meet their basic needs. This includes walking, climbing stairs, eating, personal hygiene, dressing and using the toilet. The most commonly used indices were published by Katz et al. in 1963 [1] and by Mahoney and Barthel in 1965 [5].

iADLs encompass broader areas of daily living that pose more complex challenges. These include tasks such as using the telephone, shopping, managing finances and day-to-day administrative matters, taking medication and using transport. iADLs are measured using a score based on work published by Lawton and Brody in 1969 [2].

The results are presented as totals or stratified by sex, age and education group, showing prevalences with 95% confidence intervals (95% CI). The precision of prevalences can be estimated based on 95% confidence intervals (95% CI). A wide 95% CI indicates greater statistical uncertainty of the results. Deviations of the estimated prevalence for Germany from the EU average are used to calculate statistically significant differences. A statistically significant difference between groups can be assumed if the corresponding p-value is smaller than 0.05.

**Figure 1:**
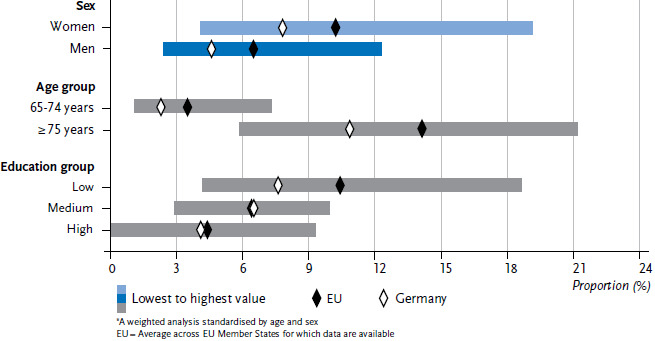
Limitations in basic activities of daily living (ADL) by sex, age and education status (n=45,197 women, n=33,817 men)* Source: EHIS 2 (2013-2015)

**Figure 2:**
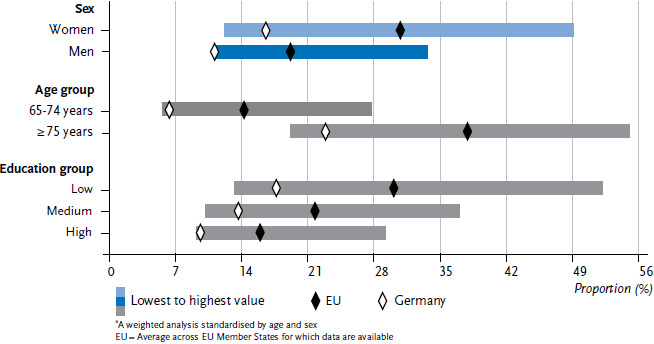
Limitations in instrumental activities of daily living (iADL) by sex, age and education status (n=45,230 women, n=33,824 men)* Source: EHIS 2 (2013-2015)

In order to provide a clear overview of the indicators, the individual values that were calculated for each of the 28 EU Member States are not set out in Figures 1 or Figure 2. Instead, the figures provide the lowest and highest values from the Member States, the EU average for the countries under consideration, and the prevalence for Germany.

The analyses were performed with a weighting factor to account for the relative population size of each EU Member State. The data are stratified by age and sex, and the study uses the European Standard Population (ESP) in its 2013 revised form. Prevalences have also been stratified by education group, with prevalences for each education group standardised by age. This improves the comparability of health indicators [23] in the Member States by accounting for possible differences in age structure. The household indicator is used as the cluster variable in the following analyses.

## Results and discussion

On average, 8.4% of people aged 65 or above in the EU report an ADL limitation and 25.2% report an iADL limitation in EHIS 2 (Table 1). Prevalences vary widely among the Member States (with ADL limitations ranging from 3.3% in Denmark to 15.3% in Belgium; and iADL limitations ranging from 11.8% in Sweden to 38.8% in Latvia). In Germany, prevalences are below the EU average (ADL limitation 6.3%, iADL limitation 14.0%).

Women are more frequently affected by ADL and iADL limitations than men (Table 1, Figure 1 and Figure 2). This also applies to Germany, albeit to a lesser extent. Men in Germany have the lowest prevalence of iADL limitation in the EU.

However, the prevalences of ADL or iADL limitations in the upper age group (75 or above) are increasing both in the EU as a whole and in Germany (Figure 1 and Figure 2). Wide variation with regard to age is also found across Member States. Prevalences for Germany remain below the EU average.

Education differences are also identified for ADL and iADL limitations (Figure 1 and Figure 2): ADL and iADL limitations are reported more frequently by people in the lower education group in Germany and across all Member States, with decreasing prevalences in higher education groups. The prevalences of ADL and iADL limitations differ among education groups within Member States with a wide variation across the Member States. The prevalence of ADL limitation for medium and high education groups in Germany is below the EU average, whereas the prevalence of iADL limitation in Germany is below the EU average for all education groups.

As previous studies have shown [10, 24, 25], the prevalences of ADL and iADL limitations differ widely between European countries despite the fact that harmonised instruments have been used to measure these indicators. People in Germany report an ADL or iADL limitation for EHIS 2 less frequently than the EU average.

**Info box 2:**

**European Health Interview Survey (EHIS)**

The European Core Health Indicators (ECHI) were jointly developed by EU Member States and international organisations, taking into account scientific and health policy requirements. The indicators provide a framework in European health reporting for population-based health surveys and analyses, and health care provision at the European and national level. The European Health Interview Survey (EHIS) is a key element in this regard. The first EHIS wave (EHIS 1), which was not mandatory, was conducted between 2006 and 2009. 17 Member States and two non-EU countries participated in EHIS 1. Participation in the second wave of EHIS (EHIS 2), which was conducted between 2013 and 2015 in all EU Member States (as well as in Iceland, Norway and Turkey) was legally binding and is based on Commission Regulation (EU) No 141/2013 of 19 February 2013. It provides essential information about the ECHI indicators. In Germany, EHIS is carried out as part of health monitoring at the Robert Koch Institute. During the EHIS 2 survey period, the EU had 28 Member States.

Further information is available at: https://ec.europa.eu/eurostat/web/microdata/european-health-interview-survey

The EHIS 2 study is the first to provide self-reported limitations in activities of daily living for people aged 65 or above living in private households for an EU-wide comparison. However, the study faces the limitation that although the EHIS 2 questionnaire was largely harmonised [15], permissible methodological differences did occur during sampling, participant acquisition and in the methods used for data collection [18]. For example, not all countries permitted the use of proxy interviews, and, in cases where proxy interviews were permitted, the option was not used to the same rate. This may have led to differences in the extent to which some countries were able to reach people aged 65 or above with severely impaired health and limitations in independent living. Moreover, these methodological differences may have contributed to the wide variation in prevalences identified in ADL and iADL limitations throughout the EU [26]. Second, due to the survey’s cross-sectional design descriptive results presented here should not be used to draw conclusions about the causes of ADL or iADL limitations, nor should they be used in attempts to explain, for example, whether gender-based differences in conducting daily activities are due to biological differences or gender roles [27, 28]. Third, iADL limitations are particularly dependent on the structure of outpatient care and the services available in a specific country. However, as the study used self-reported indicators, no data were collected on these factors. More objective indicators, such as those associated with frailty measurements, may be more appropriate to assess the heterogeneous state of the health of older community-dwelling people [29, 30]. Finally, an additional age group over 80 or 85 would also have been desirable for a better overall presentation of the results. However, data protection requirements led countries to provide information on age by different age groups; as such, a breakdown of the older age group was not available from all countries.

Nonetheless, the data from EHIS 2 constitute an important source of information for national and European health policies [15]. They provide estimations of care structures and support services that older people require and of how accessible these structures and services are to this population group. Furthermore, data from EHIS 2 also enable analyses of existing social inequalities and, thus, provide a basis with which to enact EU policies [31, 32]. In line with other countries [24, 25], Germany needs to improve prevention and care services, particularly for women, people aged 75 or over, and for people from lower education groups.

## Key statements

- The prevalences of limitations in activities of daily living vary widely between EU Member States.
- The prevalences of limitations in activities of daily living are lower in Germany than the EU average.
- Women report more often limitations in activities of daily living than men, and people aged 75 or above report more often limitations in activities of daily living than those aged between 65 and 74.
- People in the lower education group have higher prevalences of limitations in activities of daily living compared with those from higher education groups.

## Figures and Tables

**Table 1:** Prevalence of ADL and iADL limitations (standardised by age and sex) as totals and by sex and EU Member States Source: EHIS 2 (2013-2015)

	ADL limitation^1^						iADL limitation^2^					
	Women (n=45,197)		Men (n=33,817)		Total (n=79,014)		Women (n=45,230)		Men (n=33,824)		Total (n=79,054)	
	%	(95% CI)	%	(95% CI)	%	(95% CI)	%	(95% CI)	%	(95% CI)	%	(95% CI)
Austria	5.9	(4.3-8.1)	2.4	(1.5-3.9)	4.2	(3.2-5.5)	21.2	(18.3-24.4)	10.4	(7.7-14.0)	15.9	(13.9-18.3)
Belgium	19.1	(15.9-22.7)	11.3	(8.8-14.3)	15.3	(13.2-17.7)	39.5	(35.4-43.7)	24.4	(20.8-28.3)	32.2	(29.3-35.3)
Bulgaria	14.9	(12.8-17.3)	12.3	(9.9-15.2)	13.6	(12.0-15.5)	40.5	(37.5-43.6)	32.7	(29.2-36.5)	26.8	(34.2-39.4)
Croatia	12.0	(9.8-14.6)	6.8	(5.0-9.2)	9.5	(8.0-11.3)	33.7	(30.3-37.3)	22.2	(18.7-26.0)	28.2	(25.6-31.0)
Cyprus	13.6	(10.5-17.4)	6.5	(4.5-9.2)	10.2	(8.3-12.5)	49.1	(44.7-53.5)	27.3	(23.2-31.7)	38.6	(35.3-41.9)
Czech Republic	11.4	(9.6-13.5)	10.2	(8.1-12.9)	10.8	(9.4-12.5)	39.5	(36.8-42.3)	30.7	(27.3-34.3)	35.2	(33.0-37.5)
Denmark	4.1	(2.9-5.9)	2.5	(1.6-4.1)	3.3	(2.5-4.5)	16.5	(14.0-19.2)	12.4	(10.1-15.1)	14.4	(12.7-16.3)
Estonia	9.7	(7.9-11.9)	6.5	(4.4-9.6)	8.3	(6.8-10.0)	28.3	(25.4-31.5)	20.7	(17.0-25.1)	24.9	(22.5-27.4)
Finland	6.8	(5.3-8.6)	4.8	(3.3-7.0)	5.8	(4.7-7.2)	17.8	(15.5-20.3)	14.7	(12.0-17.8)	16.3	(14.5-18.2)
France	9.9	(8.4-11.7)	5.9	(4.7-7.3)	8.0	(7.0-9.1)	29.5	(27.1-32.0)	15.8	(13.9-18.0)	23.0	(21.3-24.7)
**Germany^3^**	**7.8**	**(6.7-9.2)**	**4.6**	**(3.8-5.6)**	**6.3**	**(5.5-7.1)**	**16.6**	**(15.0-18.3)**	**11.2**	**(9.9-12.8)**	**14.0**	**(12.9-15.1)**
Greece	12.7	(10.9-14.7)	9.7	(7.8-11.9)	11.2	(9.9-12.7)	38.7	(36.0-41.5)	24.6	(21.7-27.8)	32.0	(29.9-34.1)
Hungary	13.0	(10.7-15.6)	8.8	(6.4-12.0)	11.0	(9.3-13.0)	39.8	(36.3-43.5)	23.4	(19.5-27.7)	32.1	(29.4-34.9)
Ireland	6.8	(5.6-8.4)	6.0	(4.7-7.8)	6.4	(5.5-7.5)	23.3	(21.1-25.7)	19.1	(16.8-21.7)	21.3	(19.6-23.0)
Italy	12.7	(11.7-13.8)	7.3	(6.4-8.4)	10.1	(9.4-10.9)	35.2	(33.6-36.8)	20.6	(19.1-22.1)	28.2	(27.0-29.3)
Latvia	11.6	(10.0-13.4)	8.0	(6.0-10.5)	10.0	(8.7-11.4)	43.1	(40.4-45.9)	33.6	(29.8-37.5)	38.8	(36.6-41.2)
Lithuania	13.2	(11.3-15.4)	8.4	(6.2-11.2)	11.0	(9.5-12.7)	39.8	(36.8-42.9)	29.6	(25.7-33.9)	35.2	(32.7-37.7)
Luxemburg	4.9	(2.8-8.5)	5.5	(3.4-8.8)	5.2	(3.6-7.5)	20.3	(15.8-25.7)	13.2	(9.9-17.5)	16.8	(13.8-20.2)
Malta	6.6	(4.6-9.2)	3.8	(2.3-6.3)	5.2	(3.9-6.9)	34.0	(30.0-38.2)	19.2	(15.6-23.5)	26.6	(23.9-29.6)
Netherlands	13.3	(11.1-15.8)	10.3	(8.3-12.9)	11.8	(10.3-13.6)	35.6	(32.4-38.9)	23.5	(20.5-26.7)	29.6	(27.4-31.9)
Poland	10.7	(9.5-12.0)	9.0	(7.6-10.7)	9.9	(9.0-10.9)	40.7	(38.8-42.7)	27.8	(25.6-30.1)	34.5	(33.0-36.1)
Portugal	13.3	(11.6-15.3)	6.6	(5.3-8.2)	10.2	(9.0-11.4)	46.2	(43.7-48.8)	14.4	(12.5-16.5)	31.3	(29.6-33.1)
Romania	6.6	(5.6-7.8)	5.2	(4.1-6.4)	5.9	(5.2-6.8)	38.5	(36.4-40.7)	29.4	(27.1-31.7)	34.1	(32.4-35.8)
Slovakia	12.0	(9.7-14.7)	10.8	(7.8-14.6)	11.4	(9.5-13.6)	43.1	(39.5-46.8)	33.7	(29.0-38.7)	38.6	(35.6-41.6)
Slovenia	11.5	(9.3-14.1)	9.1	(6.5-12.7)	10.3	(8.5-12.4)	34.7	(31.4-38.3)	25.0	(21.3-29.2)	30.0	(27.4-32.6)
Sweden	4.9	(3.3-7.3)	3.1	(1.9-5.0)	4.0	(2.9-5.4)	12.2	(9.6-15.4)	11.4	(9.0-14.4)	11.8	(10.0-14.0)
Spain	13.4	(12.2-14.7)	7.8	(6.6-9.1)	10.6	(9.8-11.6)	40.3	(38.4-42.2)	23.3	(21.5-25.3)	32.0	(30.7-33.4)
United Kingdom	6.8	(6.0-7.8)	3.8	(3.1-4.6)	5.4	(4.8-6.0)	23.1	(21.5-24.7)	14.3	(12.9-15.8)	18.8	(17.7-19.9)
**EU**	**10.2**	**(9.8-10.6)**	**6.5**	**(6.2-6.9)**	**8.4**	**(8.2-8.7)**	**30.8**	**(30.2-31.3)**	**19.2**	**(18.7-19.8)**	**25.2**	**(24.8-25.6)**

CI = Confidence interval, EU=Average across EU Member States for which data are available

^1^ ADL limitation = At least a lot of difficulty in at least one of five ADLs (activities of daily living)

^2^ iADL limitation=At least a lot of difficulty in at least one of seven iADLs (instrumental activities of daily living)

^3^ Statistically significant differences in ADL limitations: total for Germany vs EU (p<0.001), women in Germany vs EU (p<0.01), men in Germany vs EU (p<0.01);

Statistically significant differences in iADL limitations: total for Germany vs EU (p<0.001), women in Germany vs EU (p<0.001), men in Germany vs EU (p<0.001)
