# Corrigendum: Limitations in activities of daily living in old age in Germany and the EU – Results from the European Health Interview Survey (EHIS) 2

**DOI:** 10.25646/6806

**Published:** 2020-02-03

**Authors:** B Gaertner, MA Busch, C Scheidt-Nave, J Fuchs

Gaertner B, Busch MA, Scheidt-Nave C, Fuchs J (2019)

Journal of Health Monitoring 4(4): 48-56.

DOI 10.25646/6226

In the original version of the article, on pages 48 and 50, the prevalence of iADL limitation among men in Germany was reported instead of the prevalence of iADL limitation for Germany as a whole: ‘However, prevalences vary widely between EU Member States and are lower in Germany than the EU average (ADL limitation 6.3%, iADL limitation 11.2%)’ and ‘In Germany, prevalences are below the EU average (ADL limitation 6.3%, iADL limitation 11.2%)’. The correct sentences read: ‘However, prevalences vary widely between EU Member States and are lower in Germany than the EU average (ADL limitation 6.3%, iADL limitation 14.0%)’ and ‘In Germany, prevalences are below the EU average (ADL limitation 6.3%, iADL limitation 14.0%)’.

The corrected version of the article is available at DOI 10.25646/6226.2

